# Dickkopf-1 and cytoskeletal protein 4 mRNA expression associated with liver and kidney transplant rejection: Prospective observational study

**DOI:** 10.1097/MD.0000000000044271

**Published:** 2025-09-05

**Authors:** Necip Altundaş, Eda Balkan, Murat Kizilkaya, Murat Altunok, Nurhak Aksungur, Salih Kara, Elif Demirci, Gürkan Öztürk

**Affiliations:** a Department of General Surgery, Faculty of Medicine, Atatürk University, Erzurum, Türkiye; b Department of Medical Biology, Faculty of Medicine, Atatürk University, Erzurum, Türkiye; c Department of Medical Biology, Faculty of Medicine, Ağri İbrahim Çeçen University, Ağri, Turkey; d Department of Nephrology, Trabzon Kanuni Training and Research Hospital, Trabzon, Türkiye; e Department of Pathology, Faculty of Medicine, Atatürk University, Erzurum, Türkiye.

**Keywords:** cytoskeletal protein 4, Dickkopf-1, kidney transplant rejection, liver transplant rejection, mRNA expression

## Abstract

Rejection following liver and kidney transplantation remains a major barrier to long-term graft survival. Early and reliable detection of rejection is crucial for optimizing patient outcomes and guiding personalized therapeutic approaches. Despite ongoing efforts, currently available serum-based biomarkers often fail to provide sufficient sensitivity and specificity for early diagnosis. Dickkopf-1 (DKK1) and cytoskeleton-associated protein 4 (CKAP4) are molecules involved in Wnt signaling, immune regulation, fibrosis, and tissue remodeling. Their upregulation has been associated with inflammatory and fibrotic processes in various pathological contexts. These properties make them strong candidates as novel molecular biomarkers in transplant rejection. This prospective observational study aimed to investigate the association between DKK1 and CKAP4 mRNA expression levels and the occurrence of rejection in liver and kidney transplant recipients. Peripheral blood samples from 55 transplant patients diagnosed with rejection (30 kidney, 25 liver) and 35 healthy controls were analyzed for DKK1 and CKAP4 mRNA expression using real-time polymerase chain reaction. Expression profiles were evaluated in relation to clinical and histopathological parameters. DKK1 and CKAP4 mRNA expression levels were significantly elevated in transplant recipients with rejection compared with healthy controls. In kidney transplant patients, both markers showed increased expression, although no significant histopathological correlations were detected. In liver transplant recipients, DKK1 expression was significantly associated with cellular rejection and portal inflammation. These findings suggest that DKK1 and CKAP4 may serve as promising molecular biomarkers for transplant rejection monitoring. In particular, DKK1 may provide additional diagnostic value in identifying cellular rejection and portal inflammation in liver grafts. Further multicenter studies are required to validate these results and assess their potential for clinical application.

## 1. Introduction

Kidney and liver transplantation are of vital importance in the treatment of end-stage renal disease (ESRD) and end-stage liver disease (ESLD). However, the development of posttransplant rejection directly affects graft function and patient prognosis, leading to serious complications. Transplant rejection occurs when the recipient’s immune system perceives the transplanted organ as foreign and mounts an immune-mediated response against it.^[1,2]^

ESRD is a major public health issue affecting millions of people worldwide. In the United States, approximately 750,000 new ESRD cases are reported annually. The most common causes of ESRD include diabetes and hypertension. Similarly, ESLD has an increasing prevalence, with major causes including viral hepatitis (HBV, HCV), alcoholic liver disease, nonalcoholic fatty liver disease (NAFLD), and autoimmune hepatitis.^[1,2]^ ESRD and ESLD are associated with significant morbidity and mortality globally. While ESRD typically results from the progression of chronic kidney disease (CKD) and often requires dialysis or kidney transplantation (KT), ESLD may arise due to cirrhosis, liver failure, or hepatocellular carcinoma (HCC).^[3,4]^ The most common etiological factors leading to kidney and liver failure include diabetes, hypertension, chronic viral hepatitis, nonalcoholic steatohepatitis (NASH), autoimmune diseases, and genetic or metabolic disorders. The progression of these diseases causes irreversible organ dysfunction, increasing the need for transplantation.^[3,4]^ KT improves long-term survival in patients with ESRD, while liver transplantation remains the standard treatment for both acute and chronic liver failure. Transplant recipients typically receive immunosuppressive therapy to prevent rejection. Tacrolimus-based regimens are widely used in both kidney and liver transplant recipients, often in combination with mycophenolate mofetil and steroids to reduce the risk of rejection.^[5–7]^

In recent years, Dickkopf-1 (DKK1) and cytoskeleton-associated protein 4 (CKAP4) have emerged as molecules of interest for their roles in organ fibrosis, inflammation, and immune regulation. DKK1, an inhibitor of the Wnt/β-catenin signaling pathway, plays a crucial role in tissue regeneration, inflammatory control, and modulation of immune responses.^[8]^ Animal models have demonstrated that elevated DKK1 expression aggravates tubulointerstitial fibrosis in CKD and accelerates renal function decline.^[9,10]^ Similarly, clinical studies have shown that DKK1 levels are significantly upregulated in patients with advanced liver fibrosis and cirrhosis.^[11,12]^ CKAP4, on the other hand, is involved in cellular adhesion, motility, and signal transduction, playing a vital role in maintaining tissue integrity and regulating inflammatory responses.^[13]^ Experimental models of renal failure have indicated that increased CKAP4 expression contributes to the severity of inflammation and promotes fibrotic remodeling.^[14]^ Furthermore, liver biopsies from patients with NASH and viral hepatitis have shown that higher CKAP4 levels correlate with advanced stages of fibrosis.^[14]^

Despite growing evidence of their involvement in organ fibrosis and immune modulation, the specific roles of DKK1 and CKAP4 in the context of graft rejection after kidney and liver transplantation remain largely unexplored. Given DKK1’s potential to modulate immune responses and CKAP4’s role in cellular organization, monitoring their expression levels could help elucidate the mechanisms underlying graft rejection and identify new biomarkers for posttransplant immune monitoring.^[9,15]^ Based on this background, this prospective observational study investigates the association between DKK1 and CKAP4 mRNA expression levels and the development of rejection in kidney and liver transplant recipients. We hypothesized that DKK1 and CKAP4 expression levels would be significantly elevated in patients experiencing rejection, particularly in relation to histopathological indicators of inflammation and fibrosis. This study may contribute to a better understanding of the mechanisms of rejection in kidney and liver transplantation by investigating DKK1 and CKAP4 as potential biomarkers of organ transplant rejection. We hypothesized that DKK1 and CKAP4 expression levels would be significantly elevated in patients experiencing rejection, particularly in relation to histopathological indicators of inflammation and fibrosis.

## 2. Materials and methods

This prospective observational single-center study was conducted from January to May 2025, in collaboration with the Departments of Organ Transplantation, Nephrology, Medical Biology, and Pathology at Atatürk University Faculty of Medicine. A total of 55 patients diagnosed with kidney or liver transplantation rejection (30 patients with kidney rejection and 25 patients with liver rejection) were included in this study. Both early- and late-stage rejection cases were included in this study. In the kidney transplant group, patients who developed acute rejection, typically cellular and sometimes humoral, within the first 6 months were enrolled based on clinical and laboratory findings confirming organ rejection. Similarly, in the liver transplant group, acute cellular (hepatocellular) rejection was more commonly observed within the first 6 months, and these patients were included accordingly. All participants were enrolled consecutively according to predefined inclusion and exclusion criteria to ensure the formation of a comparable control group and minimize potential confounding factors.

The inclusion criteria were as follows: patients aged ≥ 18 years who underwent kidney or liver transplantation from January to May 2025; were diagnosed with rejection based on clinical, laboratory, and histopathological findings; and agreed to participate. Patients with active infections, systemic diseases that could affect immune response, or incomplete clinical or histopathological data were excluded. Blood samples were collected prior to immunosuppressive therapy administration to avoid potential confounding effects on mRNA expression levels. Peripheral blood samples were obtained from each patient, and DKK1 and CKAP4 mRNA expression levels were quantified using real-time quantitative polymerase chain reaction (qPCR). The expression results were compared with relevant histopathological and clinical parameters.

This study was conducted in accordance with the ethical principles embodied in the Declaration of Helsinki and was approved by the Ethics Committee of Atatürk University Faculty of Medicine (ethics committee approval no.: B30.2 ATA-0.01.00/62-2025). Written informed consent was obtained from all participants.

### 2.1. Inclusion and exclusion criteria

This study included kidney and liver transplant recipients who developed rejection within 1 week to 6 months of transplantation. Blood samples were collected as part of a regular follow-up protocol prior to the initiation of immunosuppressive therapy to prevent potential drug-related effects on mRNA expression levels. Demographic and clinical data, including age, sex, type of transplantation (cadaveric or living donor), laboratory findings, type and timing of rejection, and immunosuppressive treatment regimens, were systematically recorded and carefully reviewed. This approach minimized potential differences and biases, ensured a comparable control group, and allowed for a more detailed investigation of the roles of DKK1 and CKAP4 expression in rejection mechanisms, thereby enhancing the reliability of the study.

The inclusion criteria were as follows: patients aged ≥ 18 years who underwent kidney or liver transplantation from January to May 2025; developed rejection within 1 week to 6 months after transplantation, as confirmed by clinical, laboratory, and histopathological findings; were not pregnant; and provided written informed consent.

The exclusion criteria were as follows: pregnant women, individuals aged < 18 years, those with active infections or systemic diseases that could affect the immune response, patients with missing or unclear clinical or histopathological records, those without biopsy samples, patients who had experienced graft loss, and those who died. These criteria were established to ensure data accuracy and minimize potential confounding factors.

### 2.2. Study population and data collection

The participants in this study were individuals who experienced rejection at various stages after transplantation. Complete blood counts were collected from these patients as part of a regular follow-up protocol, and demographic data (age, sex, and type of transplantation [cadaveric or living donor]) and clinical information (laboratory results, type and timing of rejection, and immunosuppressive treatment regimen) were recorded in detail.

In the kidney rejection patient group, patients who developed cellular rejection were initially treated with corticosteroids in accordance with the European Society for Organ Transplantation and Kidney Disease: Improving Global Outcomes guidelines. If necessary, calcineurin inhibitors such as tacrolimus and cyclosporine were added. In cases where no response to the initial steroid treatment was achieved, the dosage was increased. For patients who developed humoral rejection, antibodies were removed through plasmapheresis according to the American Society for Apheresis guidelines. Additionally, intravenous immunoglobulin therapy was administered, and rituximab was used when necessary.

In liver transplant patients, steroids were widely used for rejection treatment; however, the treatment approach varied depending on the etiology of the disease. In patients with autoimmune hepatitis, steroid therapy was administered in accordance with the European Association for the Study of the Liver guidelines, in which the immunosuppression protocol was adjusted in combination with antiviral treatment according to the American Association for the Study of Liver Diseases guidelines. Individualized treatment approaches were applied according to the International Liver Transplantation Society guidelines for Wilson disease and other metabolic causes. Blood samples were collected for clinical evaluation from patients who developed rejection before treatment initiation.

### 2.3. Control group

The control group comprised 35 healthy individuals with no history of kidney or liver disease, organ transplantation, autoimmune disorders, or active infections. The controls were selected to match the patient groups with respect to age and sex. The inclusion criteria for the controls were as follows: normal renal and hepatic function test results, absence of chronic systemic disease, and no current use of immunosuppressive or anti-inflammatory medications. These criteria were defined to minimize confounding variables and ensure the reliability and validity of comparative results.

### 2.4. Specimen collection

Peripheral blood samples (5–10 mL) were collected between January and May 2025 from patients who developed rejection within 1 week to 6 months after transplantation. Blood was drawn by venipuncture on the day when rejection was clinically suspected or biopsy-confirmed. The sampling protocol was applied consistently for both kidney and liver transplant recipients. Samples were collected into EDTA tubes for plasma analyses and serum separator tubes for serum analyses. To minimize circadian variation, all blood samples were obtained in the morning after overnight fasting. To preserve sample integrity and ensure reliable measurements, all samples were processed according to standard operating procedures within 30 minutes of collection and centrifuged at 3000 rpm for 10 minutes at 4°C. The resulting plasma and serum fractions were aliquoted under sterile conditions and stored at −80°C until analysis. To maintain consistency and measurement accuracy, all analyses were performed in duplicate. All collection and processing steps were carried out under controlled conditions and strictly adhered to established protocols to ensure accuracy, reliability, and reproducibility of results.

### 2.5. Technique

In this study, the clinical and laboratory parameters of kidney transplant patients who experienced rejection were prospectively recorded. In cases of suspected rejection, kidney biopsies were performed, and relevant protocols were applied for pathological evaluation. The biopsy results correlated with the diagnoses of acute cellular and humoral rejection according to the Banff 2017 criteria (Banff 2017*, Journal of Transplantation*). Acute cellular rejection was characterized by significant inflammation and cellular infiltration in tubulointerstitial areas, whereas humoral rejection was associated with vascular lesions related to the presence of antibodies.

Liver transplant recipients were prospectively monitored for rejection. When rejection was suspected, liver biopsies were performed in accordance with the relevant pathological protocols. Both cellular and humoral rejection processes were assessed based on the Banff 2017 criteria. Cellular rejection was marked by inflammation in the portal areas, blood vessel damage, and hepatocyte necrosis, whereas humoral rejection was identified by changes in the vasculature and damage associated with the presence of antibodies.

### 2.6. Analysis of DKK1 and CKAP4 gene expression: quantitative real-time PCR approach

Real-time polymerase chain reaction (real-time PCR) was performed to determine the expression levels of DKK1 and CKAP4. A series of optimization procedures were applied to enhance the accuracy and reliability of the test. ribonucleic acid (RNA) was isolated using the NucleoGene QuickEx Total RNA extraction kit, and cDNA synthesis was carried out using the NucleoGene reverse transcription kit. The PCR amplification conditions were optimized to ensure an accurate replication of the genetic material. In particular, the denaturation time, primer annealing temperature, and number of amplification cycles were adjusted to minimize error rates and maximize sensitivity. Additionally, both negative and positive control samples were included in each test to ensure the reliability of results.

### 2.7. Total RNA isolation procedure

Total RNA was isolated the NucleoGene QuickEx Total RNA Extraction Kit (Istanbul, Turkey) following the protocol recommended by the manufacturer. A 250 µL blood sample was collected and mixed with 750 µL of lysis buffer, followed by vortexing. The samples were incubated at room temperature for 10 minutes. After incubation, the samples were centrifuged at 14,000 × g for 3 minutes, and the supernatant was carefully transferred to a new tube. Subsequently, 100 µL of chloroform was added to the samples, and the mixture was left at room temperature for 3 minutes. The samples were then centrifuged again at 14,000 × g for 15 minutes. The clear upper phase was carefully transferred to a new tube, and an appropriate amount of 96% to 100% ethanol was added before vortexing. Afterward, the mixture was transferred to a spin column and centrifuged at 11,000 × g for 30 seconds; 400 µL of Wash Buffer I was then added to the column and centrifuged at 11,000 × g for 30 seconds. Next, 700 µL of Wash Buffer II was added, and the column was centrifuged at 14,000 × g for 1 minute. Finally, 50 to 100 µL of elution buffer was added, and the RNA was eluted. Prior to qPCR analysis, the purity and integrity of the isolated total RNA were assessed using a NanoDrop 2000 spectrophotometer (Thermo Scientific, USA). Samples with an A260/A280 ratio between 1.8 and 2.0 were considered acceptable for downstream applications. RNA integrity was confirmed by 1% agarose gel electrophoresis.

### 2.8. cDNA synthesis and qPCR synthesis protocol

In this study, complementary deoxyribonucleic acid (cDNA) synthesis was performed using the NucleoGene cDNA synthesis kit (Istanbul, Turkey) following the protocol recommended by the manufacturer. A mixture comprising 4 µL of NucleoGene cDNA Synthesis Kit (5x), 6 µL of RNase/DNase-free water, and 10 µL of RNA template was prepared. This mixture was amplified in a PCR program under the following conditions: at 25°C for 5 minutes, at 50°C for 30 minutes, and at 85°C for 5 minutes. Upon completion of PCR, the resulting cDNA was diluted at 1:5 and prepared for qPCR.

To detect gene expressions, qPCR analysis was conducted using the NucleoGene Gene Expression Kit (Istanbul, Turkey) following the protocol recommended by the manufacturer. A mixture containing 5 µL of OligoMix, 10 µL of SYBR Green Master Mix, 4 µL of cDNA template, and 1 µL of RNase-free water was prepared. Each sample was prepared in duplicate, and the qPCR program was set to 1 cycle at 95°C for 15 minutes, followed by 40 cycles at 95°C for 15 seconds and 60°C for 60 seconds. Readings were recorded using the SYBR Green channel.

### 2.9. Data analysis

The expression levels of DKK1 and CKAP4 genes were determined by calculating ΔCT (delta cycle threshold) values. CT represents the number of cycles required for the fluorescence to exceed a defined background threshold. ΔCT values were compared between the patient and control groups. Gene expression levels were normalized to those of GAPDH, which served as an internal control to account for variations in RNA input and cDNA synthesis efficiency.

### 2.10. Technical replicates

Each sample was tested in duplicate to ensure consistency and reliability. The same PCR procedure was applied to the control group samples for comparative analysis.

### 2.11. Statistical analysis

Data were analyzed using IBM Statistical Package for the Social Sciences (SPSS, Chicago) software version 23. The normality of distribution was assessed using the Shapiro–Wilk test. The distribution of categorical variables by group was examined using Pearson chi-squared test. Normally distributed variables were compared between groups using one-way analysis of variance (ANOVA), and correction for the false discovery rate was applied to adjust for multiple comparisons. Non-normally distributed variables were compared between groups using the Kruskal–Wallis test, and pairwise comparisons were performed using the Dunn test. The cutoff values for the variables determining kidney and liver diseases were established through receiver operating characteristic curve analysis. The relationships between normally distributed variables were evaluated using Pearson correlation coefficient, whereas the relationships between non-normally distributed variables were assessed using Spearman rho correlation coefficient. The analysis results are presented as frequencies (percentages) for categorical variables and as mean ± standard deviation or median (minimum–maximum) for quantitative variables. Statistical significance was set at *P* < .05

## 3. Results

The mean patient age was 35.3 ± 5.98 years in the control group, 41.57 ± 13.03 years in kidney transplant group, and 45.44 ± 13.74 years in the liver transplant group. The age distribution showed a statistically significant difference among the groups (*P* = .003). The sex distribution also differed significantly among the groups (*P* = .002); while males accounted for 60% in both the control and kidney transplant groups, the liver transplant group had a higher proportion of males (80%). Detailed descriptive statistics are presented in Table 1.

**Table 1:** Demographic and clinicopathologic features of kidney and liver transplant patients with rejection.

		Groups			Test statistics	*P*
		Control	Kidney patients	Liver patients		
Age	–	35.3 ± 5.98†	41.57 ± 13.03†	45.44 ± 13.74†	6719	**.003** *
		35 (26–44)	41.5 (15–60)	50 (22–65)		
		14.12 (12.35–15.43)	18.63 (16.08–21.05)	23.26 (20.35–28.46)		
Sex	Male	21 (%60)	18 (%60)	16 (80)	–	**.002** *
	Female	14 (%40)	12 (%40)	4 (20)		
mRNA DKK1		27.43 ± 1.42	22.07 ± 1.78	32.56 ± 2.14	62.941	**<.001** **
		26.77 (25.96–29.83)†	22.28 (19.36–26)†	32.79 (25.6–35.57)†		
mRNA CKAP4		17.58 ± 0.98	25.46 ± 1.26	31.25 ± 2.54	61.815	**<.001** **
		17.56 (15.71–19.69)†	25.25 (23.36–27.83)†	31.35 (20.07–34.05)†		
Creatine before rejection (mg/dL)		–	6.82 ± 3.11	–	–	–
		–	7.05 (1–13)	–		
Creatine after rejection (mg/dL)		–	3.89 ± 2.96	–	–	–
		–	2.3 (1–13)	–		
eGRF (mL/dk/1.73 m²)			33.52 ± 28.89	–	–	–
		–	39 (0–71)	–		
CRP (mg/L)		–	17.2 ± 17.36	–	–	–
		–	7.35 (2–59)	–		
Urea (mg/dL)		–	105.33 ± 51.53	–	–	–
		–	91.01 (35–210)	–		
Na (mmol/L)		–	136.74 ± 6.17	–	–	–
		–	137 (114–152)	–		
K (mmol/L)		–	4.42 ± 0.75	–	–	–
		–	4.23 (3–6)	–		
Bun (mg/dL)		–	49.09 ± 23.85	–	–	–
		–	42.53 (16–96)	–		
Interstitial inflammation (i)		–	1.33 ± 0.58	–	–	–
		–	1 (1–3)	–		
Tubulitis (t)		–	1.52 ± 0.51	–	–	–
		–	2 (1–2)	–		
Gromerulitis (q)		–	1.4 ± 0.74	–	–	–
		–	1 (1–3)	–		
Peritubular capillaritis (ptc)		–	1.5 ± 0.76	–	–	–
		–	1 (1–3)	–		
C4d		–	1.91 ± 0.7	–	–	–
		–	2 (1–3)	–		
Interstitial fibrosis (ci)		–	0.31 ± 0.17	–	–	–
		–	0.3 (0–1)	–		
ALP (40–130 U/L)		40–130 U/L 85 ± 25)	–	155.41 ± 84.22	–	–
		–	–	143.6 (63–391)		
AST (5–40 U/L)		5–40 U/L 22 ± 7)	–	230.24 ± 698.35	–	–
		–	–	23.6 (8–2550)		
ALT (5–45 U/L)		–	–	116.32 ± 269.53	–	–
		–	–	33 (7–1005)		
Total bilirubin (0.1–1.2 mg/dL)		–	–	2.43 ± 4.08	–	–
		–	–	0.95 (0–15)		
Diluted bilirubin (0.0–0.3 mg/dL)		–	–	1.24 ± 2.64	–	–
		–	–	0.22 (0–9)		
Class l MFI		–	–	1103.91 ± 1258.83	–	–
		–	–	489.5 (125–4383)		
Class ll MFI		–	–	1210.27 ± 1277.37	–	–
		–	–	666.5 (123–5477)		
Severity of portal inflammation		–	–	2.38 ± 1.03	–	–
		–	–	2 (1–4)		
Lobular inflammation		–	–	2.36 ± 0.81	–	–
		–	–	3 (1–3)		
Fibrosis		–	–	3.42 ± 1.17	–	–
		–	–	3 (2–5)		

ALP = alkaline phosphatase, ALT = alanine aminotransferase, AST = aspartate aminotransferase, BUN = blood urea nitrogen, CRP = C-reactive protein, K = potassium, MFI = mean fluorescence intensity, Na = sodium.

* One-way analysis of variance.

** Kruskal–Wallis test; mean ± standard deviation; median (minimum—maximum).

† There is no difference between AUC values with the same letter.

In our study, hypertension (40%), diabetes (46.7%), and glomerulonephritis (16.7%) were the main underlying diseases leading to ESRD in patients who underwent KT. In liver transplant patients, HBV and HCV infections (43.3%), cirrhosis (16.7%), and NASH (6.7%) were the main diseases leading to ESLD development. Some of these patients underwent transplantation because of HBV and HCV infections, whereas others had liver failure due to alcoholic cirrhosis and metabolic diseases, including NASH.

For kidney and liver transplants, the graft age range for both organs is 18 to 50 years. In emergency situations and with cadaveric organs, the acceptable graft age range is 18 to 65 years, depending on patients’ general condition and the need for urgent transplantation. In our study, organ transplantation was performed within these age ranges.

Notable variations in the mean age and mRNA expression levels were observed across different groups. Specifically, a marked difference in the mean age was found between the control group and liver disease group (*P* = .003). However, the age of the kidney disease group did not differ significantly from that of the other groups.

Compared with those in healthy controls, the mRNA expression levels of CKAP4 were significantly elevated in both kidney and liver transplant recipients with rejection, whereas the DKK1 levels were significantly increased only in the liver transplant recipients. These findings suggest that CKAP4 may serve as a potential biomarker for kidney and liver transplant rejection (*P* < .001; Table 1).

In this study, the demographic and clinical features of the kidney and liver disease groups were examined in detail. Parameters such as sex distribution, rejection type, donor source, and HLA compatibility scores are presented in Table 2.

**Table 2:** Comparison of categorical variables by kidney and liver transplant patients with rejection.

	Groups			Test statistics	*P*
	Control	Kidney patients	Liver patients		
Sex					
Female	15 (45)	7 (24.1)	11 (44)	3143	.208*
Male	20 (55)	23 (75.9)	14 (56)		
Type of rejection					
Cellular	–	10 (33.3)	19 (64.3)	–	–
Humoral	–	20 (66.7)	7 (35.7)		
Transport method					
Live	–	18 (60)	19 (79)	–	–
Cadaver	–	12 (40)	6 (21)		
HLA matching degree~					
1A	–	13 (48.1)	–	–	–
1B	–	18 (66.7)	–		
1DR	–	16 (59.3)	–		
2A	–	1 (3.7)	–		
2B	–	1 (3.7)	–		
2DR	–	7 (25.9)	–		
DSA class l					
Negative	–	30 (100)	–	–	–
DSA class II					
Negative	–	30 (100)	–	–	–
DSA class l					
Negative	–	–	19 (73.9)	–	–
Positive	–	–	6 (26.1)		
DSA class ll					
Negative	–	–	16 (60.9)	–	–
Positive	–	–	9 (39.1)		
Type of portal inflammation					
Eosin neuroph plasma lymph	–	–	1 (7.1)	–	–
Lymphocyte	–	–	2 (14.3)	–	–
Lymphocyte plasma	–	–	1 (7.1)	–	–
plasma	–	–	1 (7.1)	–	–
Plasma eosin neutral	–	–	4 (28.6)	–	–
Plasma lymphocyte	–	–	3 (21.4)	–	–
Polymorphonuclear leukocytes	–	–	1 (7.1)	–	–
Hyperchromasia					
Positive	–	–	10 (100)	–	–
Nucleal					
Positive	–	–	10 (100)	–	–
Bile duct epithelial arrangement					
Positive	–	–	10 (100)	–	–

DSA = donor-specific antibodies, HLA = human leukocyte antigen.

* Pearson chi-square test; frequency (percentage); ~multiple responses.

**Figure 1.**
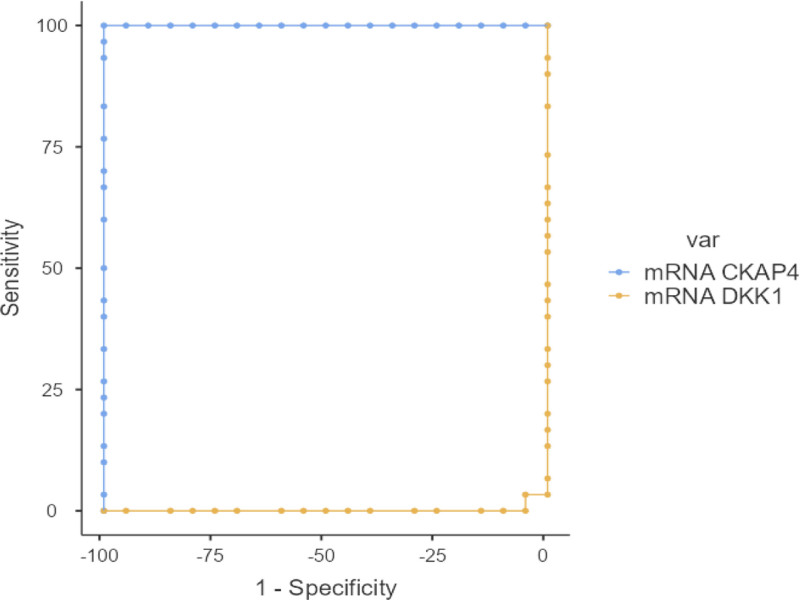
Receiver operating characteristic curve of the parameters used to distinguish kidney rejection.

**Figure 2.**
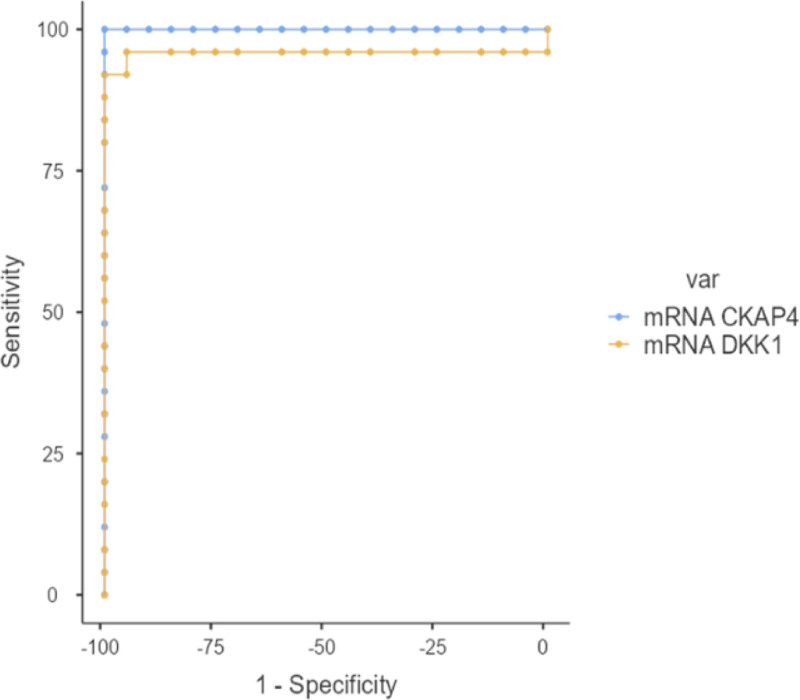
Receiver operating characteristic curve of the parameters used to distinguish liver rejection.

In our study, statistically significant cutoff values for DKK1 and mRNA CKAP4 variables were determined to distinguish kidney diseases from liver diseases (*P* < .001; Tables 3 and 4, Figs. 1 and 2).

**Table 3:** Establishing the cutoff threshold for mRNA (baseline) variables in predicting kidney rejection.

	Cutoff value	AUC (% 95 CI)	*P*	Sensitivity (%)	Specificity (%)	PPV (%)	NPV (%)
mRNA DKK1	≤24.82	0.998 (0.993–1)	<.001	93.33	100	100	90.91
mRNA CKAP4	≥23.36	1 (1–1)	<.001	100	100	100	100

**Table 4:** Establishing the cutoff threshold for mRNA (baseline) variables in predicting liver rejection.

	cutoff value	AUC (%95 CI)	*P*	Sensitivity (%)	Specificity (%)	PPV (%)	NPV (%)
mRNA DKK1	≥ 29.99	0.958 (0.881–1)	<.001	92	100	100	90.91
mRNA CKAP4	≥ 20.07	1 (1–1)	<.001	100	100	100	100

A statistically significant cutoff value was found for mRNA CKAP4 variable (*P* < .001). When the mRNA CKAP4 variable was ≥ 23.36, a sensitivity of 100%, specificity of 100%, positive predictive value (PPV) of 100%, and negative predictive value (NPV) of 100% were obtained. A statistically significant cutoff value was also found for mRNA DKK1 variable (*P* < .001). When the mRNA DKK1 variable was ≤ 24.82, a sensitivity of 93.33%, specificity of 100%, PPV of 100%, and NPV of 90.91% were obtained (Table 3, Fig. 1).

A statistically significant cutoff value was found for mRNA CKAP4 variable (*P* < .001). When the mRNA CKAP4 variable was ≥20.07, a sensitivity of 100%, specificity of 100%, PPV of 100%, and NPV of 100% were obtained. A statistically significant cutoff value was also found for mRNA DKK1 variable (*P* < .001). When the mRNA DKK1 variable was ≥ 29.99, a sensitivity of 92%, specificity of 100%, PPV of 100%, and NPV of 90.91% were obtained (Table 4, Fig. 2).

No significant correlation was observed between the mRNA levels of DKK1 and CKAP4 and the levels of interstitial inflammation (i), tubulitis (t), glomerulitis (q), peritubular capillaritis (ptc), C4d, or interstitial fibrosis (ci) in kidney rejection patients (*P* > .050) (Table 5).

**Table 5:** Analysis of the relationship between variables and histopathological findings in kidney transplant rejection patients.

		mRNA DKK1	mRNA CKAP4
Interstitial inflammation (i)	r	0.318	0.081
	p	0.160	0.728
Tubulitis (t)	r	−0.033	−0.079
	p	0.882	0.720
Glomerulitis (q)	r	0.100	−0.319
	p	0.722	0.246
Peritubular capillaritis (ptc)	r	0.185	0.410
	p	0.527	0.145
C4d	r	0.144	0.071
	p	0.674	0.837
Interstitial fibrosis (ci)	r	−0.397	−0.478
	p	0.227	0.137

r: Spearman rho correlation coefficient.

Furthermore, the mRNA levels of DKK1 and CKAP4 showed a statistically significant relationship with histopathological findings in patients after liver transplantation (*P* < .050). Specifically, the mRNA DKK1 variable was associated with portal inflammation severity in patients after liver transplantation. These results highlight the role of DKK1 in monitoring and treatment strategies following liver transplantation (Table 6).

**Table 6:** Analysis of the relationship between variables and histopathological findings in liver transplant rejection patients.

		mRNA CKAP4	mRNA DKK1
Severity of portal inflammation	r	0.095	−0.203
	p	0.727	0.025
Lobular inflammation	r	0.169	0.131
	p	0.619	0.700
Fibrosis	r	0.428	0.013
	p	0.165	0.969

r: Spearman rho correlation coefficient.

No statistically significant difference was found in the average values of mRNA DKK1 according to the type of kidney rejection (*P* = .748); the average value was 22.22 in cellular rejection and 21.99 in humoral rejection. No statistically significant difference was also detected in the average values of mRNA CKAP4 (*P* = .532); the average value was 25.67 in cellular rejection and 25.36 in humoral rejection (Table 7).

**Table 7:** Analysis of mRNA DKK1, mRNA CKAP4 expression levels according to rejection types after KT.

	Rejection type				Test statistic	*P* *
	Cellular		Humoral			
	Mean ± standard deviation	Median (min–max)	Mean ± standard deviation	Median (min–max)		
mRNA DKK1	22.22 ± 2.03	22.31 (20.13–26)	21.99 ± 1.68	22.28 (19.36–25.58)	0.324	.748
mRNA CKAP4	25.67 ± 1.43	25.45 (23.52–27.83)	25.36 ± 1.19	25.25 (23.36–27.63)	0.633	.532

* Independent samples *t*-test.

KT = kidney transplantation.

A significant difference in liver transplant rejection was observed between the cellular and humoral rejection groups in patients with liver transplant rejection according to rejection type (*P* = .042). The mRNA DKK1 expression level was 24.22 ± 3.33 in the cellular rejection group and 21.99 ± 1.68 in the humoral rejection group. These findings indicated that mRNA DKK1 expression was significantly higher in the cellular rejection group (*P* = .042; Table 8).

**Table 8:** Comparison of mRNA DKK1 and mRNA CKAP4 expression levels according to rejection types after liver transplantation.

	Rejection type				Test statistic	*P* *
	Cellular		Humoral			
	Mean ± standard deviation	Median (min–max)	Mean ± standard deviation	Median (min–max)		
mRNA DKK1	24.22 ± 3.33	23.31 (21.13–28)	21.99 ± 1.68	22.28 (19.36–25.58)	0.324	.042
mRNA CKAP4	25.67 ± 1.43	25.45 (23.52–27.83)	25.36 ± 1.19	25.25 (23.36–27.63)	0.633	.532

* Independent samples *t*-test.

## 4. Discussion

Our findings demonstrated that CKAP4 mRNA expression levels were significantly elevated in both liver and kidney tissues of patients who developed rejection after transplantation compared with healthy controls (*P* < .01).

Among kidney transplant recipients, a significant difference in the mRNA levels of CKAP4 was observed between the rejection and control groups. However, no significant increase in DKK1 levels was found, and there was no significant correlation with histopathological findings. In liver transplant recipients, the expression levels of both genes increased, with DKK1 expression particularly showing a significant association with cellular rejection and portal inflammation.

No relationship was found between DKK1 or CKAP4 expression and other histopathological features, such as fibrosis and cavernous changes. Taken together, these results suggested that CKAP4 mRNA levels may serve as a potential biomarker for monitoring transplant rejection in both kidney and liver recipients, whereas DKK1 appears to be particularly relevant for identifying cellular rejection and portal inflammation in liver transplantation.

DKK1 is a key regulator of the Wnt signaling pathway and plays a role in cellular proliferation, differentiation, and apoptosis.^[10,11]^ LRP6 binding inhibits signal transmission and this interaction is considered an important regulatory mechanism in various types of cancers and fibrotic diseases.^[11,12]^ Moreover, its similar binding affinity for CKAP4 suggests that it may exert its effects through different receptors on the cell surface.^[13,14]^ These findings indicate that DKK1 may serve as a potential biomarker and therapeutic target in pathological conditions.^[9,14]^

Overexpression of DKK1 in cancer cells promotes cell migration, proliferation, and invasion. Increased DKK1 levels have been reported in different types of cancers. However, the oncogenic and tumor suppressive effects of DKK1 on cancer cells remain unclear.

### 4.1. Findings in kidney transplant patients

Çoban et al observed that compared with healthy individuals, the development of atherosclerosis and arterial stiffness increased in renal transplant recipients; however, no difference in DKK1 levels was found between the groups concerning kidney function improvement.^[15]^ Additionally, DKK1 was determined to be not associated with atherosclerosis and arterial stiffness. Although the relationship between DKK1, vascular processes, and renal function has been previously studied, its role in transplant rejection remains unclear. In our study, we sought to address this gap by evaluating the potential role of DKK1 in kidney transplant rejection for the first time.^[15]^

Another significant finding comes from the study by Lin et al, in which DKK1 promoted mesangial matrix accumulation and kidney dysfunction induced by high glucose.^[16]^ The role of DKK1 in a high glucose environment was examined, and this molecule was determined to increase the expression of profibrotic factors such as TGF-β1 and fibronectin in mesangial cells. The study revealed that DKK1 affected the nuclear stability of β-catenin in response to high glucose and contributed to kidney dysfunction by increasing matrix accumulation in mesangial cells. Silencing DKK1 prevented kidney function impairment and microstructural changes in diabetic nephropathy. DKK1 may accelerate the progression of diabetic nephropathy, and DKK1 inhibition may be considered a therapeutic approach for this disease.^[16]^

Zhou et al reported that the inhibition of the Wnt/β-catenin signaling pathway prevented the progression to CKD and improved the kidney function. The inhibitory effects of DKK1 played crucial roles in this process, suggesting that DKK1 inhibition should be considered a new strategy for CKD treatment.^[17]^

Although the association between DKK1 and fibrosis have been reported in the literature, their effects on kidney rejection remain to be explored. Our study is one of the first investigations to evaluate the potential role of DKK1 in KT; however, we did not observe a significant association between DKK1 expression and kidney transplant rejection. This finding, when compared with existing knowledge regarding the possible effects of DKK1 on fibrosis and inflammatory processes, suggests that its role in kidney transplant rejection may be limited in this sample. Nevertheless, further studies with larger patient populations and different methodologies should be conducted to clarify the potential diagnostic or prognostic value of DKK1 in this context.

CKAP4 has been demonstrated by recent studies to be associated with the cytoskeleton and to play a significant role as a biomarker with diagnostic and therapeutic potential in various malignancies.^[18]^ CKAP4 has drawn attention owing to its role in vascular calcification and the fibrotic changes involved in kidney rejection. CKAP4 affects YAP phosphorylation and MMP2 expression. Nevertheless, there remains insufficient information regarding how CKAP4 and DKK1 interact during kidney transplant rejection. Our study aims to address this gap.^[9]^

DKK1 expression has been suggested by previous studies to be related to the regulation of tissue repair mechanisms and the modulation of chronic inflammatory processes.^[19]^ While DKK1 is thought to contribute to T cell-mediated immune responses during transplant rejection, our findings did not indicate a significant association between DKK1 expression and kidney transplant rejection in this cohort. Increased CKAP4 expression is associated with dendritic cell activation and inflammatory cytokine production.^[18]^ This increase in kidney transplant rejection suggests that pro-inflammatory signals are activated during the graft immune response. However, no significant differences in DKK1 and CKAP4 expression levels were observed between the cellular and humoral rejection subtypes, implying that the contribution of both genes to the rejection process may be more closely related to general inflammatory response mechanisms. Evaluation of additional parameters, including histological examinations, inflammatory cytokine level assessments, and immune cell profiling in larger cohorts, may aid in further clarifying the potential role of DKK1 and CKAP4 in kidney transplant rejection.

### 4.2. Findings in liver transplant patients

While discussing the relationship between liver transplant rejection and DKK1 and CKAP4 expression, it should be noted that although studies directly addressing this specific topic are limited, several important studies have investigated the effects of the DKK1–CKAP4 signaling axis in HCC.

Iguchi et al showed that the DKK1-CKAP4 signaling axis increases the aggressiveness of HCC. In their study, it was determined that DKK1 supports cell proliferation by binding to CKAP4, and blocking this axis inhibited tumor growth. High expression of both DKK1 and CKAP4 in patients with HCC has been associated with poor prognosis.^[9]^

The Wnt/β-catenin pathway plays a crucial role in regulating cell proliferation and fibrosis.^[17–19]^ Our study identified increased mRNA expression levels of both DKK1 and CKAP4 during liver rejection. This increased expression of DKK1 may suggest the suppression of the Wnt pathway, which may be associated with the inhibition of cell proliferation and enhanced fibrosis formation.

Fatima et al highlighted the importance of DKK family members as potential diagnostic and prognostic markers for HCC. DKK1 has been found to have a higher diagnostic value than serum AFP levels in HCC. In particular, in AFP-negative HCC cases, DKK1 may be more effective in differentiating HCC from nonmalignant liver diseases. This suggests that DKK1 is an important alternative in situations where AFP alone is insufficient.^[20]^

Our findings are consistent with those of previous studies investigating these pathways. DKK1 and CKAP4 mRNA expression levels were higher in patients with liver rejection than in controls. Specifically, DKK1 showed higher expression in cases of cellular rejection and was significantly associated with portal inflammation. These results suggest that DKK1 and CKAP4 may play potential roles as biomarkers of liver rejection and may have a significant impact on pathogenesis.

These findings are consistent with those reported in the literature. DKK1 and CKAP4 mRNA expression levels were higher in patients with liver rejection than in controls. Specifically, DKK1 showed higher expression in cases of cellular rejection and was significantly associated with portal inflammation. These results suggest that DKK1 and CKAP4 may play potential roles as biomarkers of liver rejection and that these molecules may have a significant impact on the pathogenesis of liver rejection. In our study, DKK1 expression increased during liver rejection, likely due to inflammation and immune activation. This upregulation may reflect Wnt/β-catenin pathway suppression, promoting cell proliferation and fibrosis. These results suggest that DKK1–CKAP4 interaction may contribute to both HCC progression and graft rejection.

In the analysis performed according to rejection type, a significant difference in mRNA DKK1 expression levels was observed between the cellular and humoral rejection groups. The mRNA DKK1 expression in the cellular rejection group is statistically significant compared than in the humoral rejection group. This finding suggests that DKK1 may be an important biomarker in the liver transplant rejection process, especially for differentiating between cellular and humoral rejection. These results may contribute to a better understanding of liver rejection and development of treatment strategies.

In summary, our study provides novel insights into the potential roles of DKK1 and CKAP4 as biomarkers in liver transplant rejection, particularly in relation to cellular rejection and portal inflammation. By demonstrating significantly increased mRNA expression in rejected liver grafts, our findings contribute to the limited existing knowledge on the molecular mechanisms underlying liver allograft rejection. Additionally, elevated levels of liver function parameters such as AST and ALP in the rejection group further support the presence of underlying inflammatory and fibrotic activities associated with graft rejection, which may have clinical implications for improving diagnostic accuracy, differentiating rejection subtypes, and guiding future research to develop targeted therapies focusing on the DKK1–CKAP4 axis.

### 4.3. Limitations

Although our study has some limitations, they may serve as important guidelines for future research. First, the lack of data collection before kidney and liver transplantation indicates that the early-stage effects of rejection should be assessed more comprehensively. Furthermore, the relatively small sample size, which included 30 kidney transplant recipients and 25 liver transplant recipients, underscores that future studies involving larger patient populations would enhance the statistical power and improve the broader applicability of the results.

Our study was a single-center study, highlighting the need for multicenter research. A better understanding of the roles of DKK1 and CKAP4 in both transplantation and organ rejection will significantly contribute to the knowledge in this field. Furthermore, the short follow-up duration emphasizes the need to investigate prolonged effects in future studies.

These limitations serve as guidelines for more comprehensive and robust studies that could increase the validity of the findings. Future research based on these initial findings may provide detailed and extensive results. Additionally, incorporating various molecular analyses, such as ELISA and western blotting, could further clarify the role of biomarkers, contributing to a better understanding of the pathophysiological mechanisms in the transplant process. To provide more information on this subject, advanced studies should be conducted to investigate the blood and tissue expression levels of these biomarkers.

Moreover, exploring genetic and epigenetic regulators, particularly miRNAs, lncRNAs, and other biomolecules, may be important to assess their effects on posttransplant immune responses and graft health. Such advanced molecular studies could help personalize patient management and contribute to the development of new strategies aimed at improving the long-term outcomes after transplantation.

## 5. Conclusion

This pilot study is one of the first to simultaneously investigate DKK1 and CKAP4 expression in patients who develop rejection after liver and KT. Our results indicate that CKAP4 mRNA levels are elevated in both kidney and liver transplant rejections, suggesting its potential as a biomarker for monitoring rejection. Among liver transplant recipients, DKK1 expression was notably increased, especially in cases of cellular rejection and portal inflammation, indicating a possible association with inflammatory responses. However, no significant increase in DKK1 levels was observed in kidney transplant recipients with rejection. Therefore, larger and more diverse cohorts as well as advanced molecular analyses are required to clarify the potential clinical utility of these biomarkers.

## Author contributions

**Conceptualization:** Necip Altundaş, Eda Balkan, Murat Kizilkaya.

**Data curation:** Eda Balkan.

**Formal analysis:** Necip Altundaş, Eda Balkan.

**Investigation:** Necip Altundaş, Eda Balkan, Murat Kizilkaya, Murat Altunok, Nurhak Aksungur, Salih Kara, Elif Demirci, Gürkan Öztürk.

**Methodology:** Necip Altundaş, Eda Balkan, Murat Kizilkaya, Murat Altunok, Nurhak Aksungur, Salih Kara.

**Resources:** Necip Altundaş, Eda Balkan, Murat Kizilkaya, Murat Altunok, Nurhak Aksungur, Salih Kara, Elif Demirci, Gürkan Öztürk.

**Software:** Eda Balkan.

**Supervision:** Necip Altundaş, Eda Balkan.

**Validation:** Necip Altundaş, Eda Balkan.

**Writing – original draft:** Necip Altundaş, Eda Balkan.

**Writing – review & editing:** Eda Balkan.

## References

[1] McCullough KP Morgenstern H Saran R Herman WH Robinson BM. Projecting ESRD incidence and prevalence in the United States through 2030. J Am Soc Nephrol. 2019;30:127–35. 30559143 10.1681/ASN.2018050531 PMC6317596

[2] Gan C Yuan Y Shen H. Liver diseases: epidemiology, causes, trends and predictions. Signal Transduct Target Ther. 2025;10:33. 39904973 10.1038/s41392-024-02072-z PMC11794951

[3] Saran R Robinson B Abbott KC. US renal data system 2019 annual data report: epidemiology of kidney disease in the United States. Am J Kidney Dis. 2020;75(1 Suppl 1):A6–7. 31704083 10.1053/j.ajkd.2019.09.003

[4] Patrick CM Barritt AS. Chronic liver disease. In Gi and Liver Disease during Pregnancy: A Practical Approach. CRC Press (Taylor & Francis Group); 2023.

[5] Raja K Panackel C. Post liver transplant renal dysfunction—evaluation, management and immunosuppressive practice. J Clin Exp Hepatol. 2024;14:101306. 38274509 10.1016/j.jceh.2023.101306 PMC10806298

[6] Kovac D Choe J Liu E. Immunosuppression considerations in simultaneous organ transplant. Pharmacotherapy. 2021;41:59–76. 33325558 10.1002/phar.2495

[7] Bauer AC Franco RF Manfro RC. Immunosuppression in kidney transplantation: state of the art and current protocols. Curr Pharm Des. 2020;26:3440–50. 32436821 10.2174/1381612826666200521142448

[8] Kimura H Fumoto K Shojima K. CKAP4 is a Dickkopf1 receptor and is involved in tumor progression. Signal Transduct Target Ther. 2016;6:e11906. 10.1172/JCI84658 PMC4922689 27322059

[9] Iguchi K Sada R Matsumoto S. DKK1-CKAP4 signal axis promotes hepatocellular carcinoma aggressiveness. Cancer Sci. 2023;114:2063–77. 36718957 10.1111/cas.15743 PMC10154837

[10] Niehrs C. Function and biological roles of the Dickkopf family of Wnt modulators. Oncogene. 2006;25:7469–81. 17143291 10.1038/sj.onc.1210054

[11] Nagoya A Sada R Kimura H. CKAP4 is a potential exosomal biomarker and therapeutic target for lung cancer progression. Transl Lung Cancer Res. 2023;12:408–26. 37057110 10.21037/tlcr-22-571 PMC10087988

[12] Klavdianou K Liossis SN Daoussis D. Wnt signaling as a therapeutic target in rheumatoid arthritis and other chronic inflammatory diseases. Mediterr J Rheumatol. 2017;28:174–82. 32185280 10.31138/mjr.28.4.174 PMC7045998

[13] Kimura H Yamamoto H Harada T. CKAP4, a DKK1 receptor, is a biomarker in exosomes derived from pancreatic cancer and a molecular target for therapy. Clin Cancer Res. 2019;25:1936–47. 30610103 10.1158/1078-0432.CCR-18-2124

[14] Shinno N Kimura H Sada R. Activation of the Dickkopf1-CKAP4 pathway is associated with poor prognosis of esophageal cancer and anti-CKAP4 antibody may be a new therapeutic drug. Oncogene. 2018;37:3471–84. 29563607 10.1038/s41388-018-0179-2

[15] Coban M Durak BA Karakan MS. Relationship of Dickkopf-1 with atherosclerosis and arterial stiffness in renal transplant recipients. Transplant Proc. 2024;56:1937–46. 39477726 10.1016/j.transproceed.2024.10.019

[16] Lin CL Wang JY Ko JY Huang YT Kuo YH Wang FS. Dickkopf-1 promotes hyperglycemia-induced accumulation of mesangial matrix and renal dysfunction. J Am Soc Nephrol. 2010;21:124–35. 20019166 10.1681/ASN.2008101059 PMC2799277

[17] Zhou G Li J Zeng T Yang P Li A. The regulation effect of WNT-RAS signaling pathway on renal fibrosis in chronic kidney disease. J Nephrol. 2020;33:289–97. 31392659 10.1007/s40620-019-00637-8 PMC7118045

[18] Li S-X Li J Dong L-W Guo Z-Y. Cytoskeleton-associated protein 4, a promising biomarker for tumor diagnosis and therapy. Front Mol Biosci. 2021;7:552056. 33614703 10.3389/fmolb.2020.552056 PMC7892448

[19] Koch S Nava P Addis C. The Wnt antagonist Dkk1 regulates intestinal epithelial homeostasis and wound repair. Gastroenterology. 2011;141:259–68, 268.e1. 21440550 10.1053/j.gastro.2011.03.043 PMC3551610

[20] Fatima S Luk JM Poon RTP Lee NP. Dysregulated expression of dickkopfs for potential detection of hepatocellular carcinoma. Expert Rev Mol Diagn. 2014;14:535–48. 24809435 10.1586/14737159.2014.915747

